# Lessons on Climate Sensitivity From Past Climate Changes

**DOI:** 10.1007/s40641-016-0049-3

**Published:** 2016-10-20

**Authors:** Anna S. von der Heydt, Henk A. Dijkstra, Roderik S. W. van de Wal, Rodrigo Caballero, Michel Crucifix, Gavin L. Foster, Matthew Huber, Peter Köhler, Eelco Rohling, Paul J. Valdes, Peter Ashwin, Sebastian Bathiany, Tijn Berends, Loes G. J. van Bree, Peter Ditlevsen, Michael Ghil, Alan M. Haywood, Joel Katzav, Gerrit Lohmann, Johannes Lohmann, Valerio Lucarini, Alice Marzocchi, Heiko Pälike, Itzel Ruvalcaba Baroni, Dirk Simon, Appy Sluijs, Lennert B. Stap, Alexis Tantet, Jan Viebahn, Martin Ziegler

**Affiliations:** 1 Institute for Marine and Atmospheric Research, Centre for Extreme Matter and Emergent Phenomena, Utrecht University, Princetonplein 5, 3584 CC Utrecht, The Netherlands; 2 Department of Meteorology and Bolin Centre for Climate Research, Stockholm University, 10691 Stockholm, Sweden; 3 Earth and Life Institute, Université catholique de Louvain, 1348 Louvain-la-Neuve, Belgium; 4 Ocean and Earth Science, University of Southampton, National Oceanography Centre Southampton, Southampton, SO14 3ZH UK; 5 Earth, Atmospheric and Planetary Sciences, Purdue University, West Lafayette, IN 47907 USA; 6 Institute for the Study of Earth, Oceans, and Space, University of New Hampshire, Durham, NH 03814 USA; 7 Alfred-Wegener-Institut Helmholtz-Zentrum für Polar-und Meeresforschung (AWI), P.O. Box 12 01 61, 27515 Bremerhaven, Germany; 8 Research School of Earth Sciences, The Australian National University, Canberra, 2601 Australia; 9 Cabot Institute and School of Geographical Sciences, University of Bristol, Bristol, BS8 1SS UK; 10 Centre for Systems, Dynamics and Control, Department of Mathematics, University of Exeter, Exeter, EX4 4QF UK; 11 Department of Environmental Sciences, Wageningen University, 6700 AA Wageningen, The Netherlands; 12 Department of Earth Sciences, Faculty of Geosciences, Utrecht University, Heidelberglaan 2, 3584 CS Utrecht, The Netherlands; 13 Niels Bohr Institute, University of Copenhagen, Juliane Maries Vej 30, 2100 Copenhagen O, Denmark; 14 Department of Geosciences and Laboratoire de Météorologie Dynamique (CNRS and IPSL), Ecole Normale Supérieure, 75231 Paris Cedex, France; 15 Department of Atmospheric and Oceanic Sciences, University of California, Los Angeles, 90095-9567 USA; 16 School of Earth and Environment, University of Leeds, Woodhouse Lane, Leeds LS2 9JT UK; 17 School of Innovation Sciences, Philosophy Department, Eindhoven University of Technology, Eeuwsel 5612, AZ Eindhoven, The Netherlands; 18 School of Historical and Philosophical Inquiry, The University of Queensland, St Lucia, QLD 4072 Australia; 19 MARUM-Center for Marine Environmental Sciences, University of Bremen, Leobener Strasse, 28359 Bremen, Germany; 20 CEN, Institute of Meteorology, University of Hamburg, Hamburg, Germany; 21 Department of Mathematics and Statistics, University of Reading, Reading, UK; 22 Department of the Geophysical Sciences, The University of Chicago, 5734 South Ellis Avenue, Chicago, IL 60637 USA; 23 Faculty of Geosciences, Utrecht University, Princetonplein 9, 3584 CC Utrecht, The Netherlands; 24 Department of Earth Sciences, Utrecht University, PO Box 80.021, 3508 TA Utrecht, The Netherlands

**Keywords:** Climate sensitivity, Palaeoclimate, Climate tipping points

## Abstract

Over the last decade, our understanding of climate sensitivity has improved considerably. The climate system shows variability on many timescales, is subject to non-stationary forcing and it is most likely out of equilibrium with the changes in the radiative forcing. Slow and fast feedbacks complicate the interpretation of geological records as feedback strengths vary over time. In the geological past, the forcing timescales were different than at present, suggesting that the response may have behaved differently. Do these insights constrain the climate sensitivity relevant for the present day? In this paper, we review the progress made in theoretical understanding of climate sensitivity and on the estimation of climate sensitivity from proxy records. Particular focus lies on the background state dependence of feedback processes and on the impact of tipping points on the climate system. We suggest how to further use palaeo data to advance our understanding of the currently ongoing climate change.

## Introduction

The concept of climate sensitivity has been introduced with the aim of providing a measure of the response of the climate system to ‘external’ perturbations to Earth’s radiative balance. It is a useful quantity for projecting climate changes over the coming century as a response to increasing concentrations of atmospheric greenhouse gases.

The simplest framework of climate sensitivity is to think of the difference in global annual mean surface temperature Δ*T* between two statistical steady states, which have a different pCO_2_ level. A general equilibrium climate sensitivity parameter, indicated here by *S*, is given by

1 $$ S = \frac{\Delta T}{\Delta R}  $$

where Δ*R* is the difference in radiative forcing between the climate states. More specifically, if Δ*R* is the radiative forcing associated with a doubling of atmospheric CO_2_, then the equilibrium climate sensitivity (ECS) is defined as ECS = $S\cdot {\Delta } R_{2xCO_{2}}$. Due to the presence of feedbacks, the radiative perturbation can be amplified to lead to a larger Δ*T* than would be expected based on the direct effect of the increase in pCO_2_ on the radiative balance [1, 2].

Observational studies using the instrumental period together with model simulations give a range of ECS values between 1.5 and 4.5 ^∘^C per CO_2_ doubling [3]. There remain, however, considerable uncertainties regarding the range of ECS, in particular concerning its upper limits, although understanding of the spread in model-based results is emerging, suggesting short-wave cloud feedbacks as the dominant source of intermodel differences [4–6]. Furthermore, analysis of ECS from the response to individual forcings [7], observational constraints from mixed-phase clouds [8], and estimates of transient climate sensitivity from observations of the last half century [9] all suggest that in particular, the higher values cannot be rejected.

Past climate changes can help to estimate the response of the climate system to variations in external forcing or greenhouse gas concentrations [10, 11]. One approach consists in considering ensembles of experiments with climate models, either perturbed parameter ensembles [12–14] or multi-model ensembles [15, 16], which may also be calibrated to past climate observations [17]. Climate sensitivity estimates with this method may be delivered as probability distributions following the paradigm of Bayesian inference, but with the usual caveats of the many assumptions on the model, the observations used, and judgements on uncertainties of models and data [18]. The posterior distribution obtained with a given climate model may differ from another one. It is also expected to depend on the choice of observations used for calibrations, as well as on judgements about model uncertainties. Finally, experts advocate the importance of careful considerations about model structural errors, often termed discrepancy in this context [19]. Another approach for estimating climate sensitivity is to use time series of past climate changes and estimate climate sensitivity relevant to the present-day problem based on differences between different times sampled within the time series [20]. As more palaeoclimate data become available, with better estimates of pCO_2_ and Δ*T*, there appears to be a strong potential for the latter approach to be successful and thereby provide an independent estimate of the present ECS.

However, the response of the climate system to a radiative forcing perturbation occurs on many different timescales, due to the presence of different feedbacks [21]. This multi-scale response becomes particularly important if long timescales associated with the geological record are considered. On a timescale of a hundred years, the effect of fast feedbacks, such as the water-vapour feedback, has equilibrated. However, slower feedbacks, such as those associated with ocean heat uptake, or even slower land-ice changes will continue to cause adjustment of the climate system.

For the present climate, the equilibrium climate sensitivity parameter *S* has been introduced with a century timescale in mind [22], indicating that only a limited number of (relatively fast acting, <100 years) feedback processes are taken into account. The radiative perturbation Δ*R* is considered as the effective radiative forcing after very fast processes have equilibrated [3]. As climate model simulations generally do not reach full equilibrium, in particular with respect to ocean heat uptake, *S* is determined from the transient towards that equilibrium using the residual net top-of-the-atmosphere radiative imbalance [23].

When slow feedbacks also affect a proxy time series, one cannot determine *S* directly from these data. To this end, the concept of Earth system sensitivity (ESS) has been introduced [24], with ESS = $S^{p}\cdot {\Delta } R_{2xCO_{2}}$ and *S* ^*p*^ (where ‘p’ stands for palaeo) quantifying the long-term (>1000 years) equilibrium response in global mean surface temperature after an increase in atmospheric pCO_2_ including the multi-scale Earth system feedbacks (except carbon cycle feedbacks). Because many more positive (slow) feedbacks are involved, *S* ^*p*^ is generally larger than *S* [24, 25].

In [21], the relation between *S* ^*p*^ and *S* was clarified and it was also described how to meaningfully compare values among different studies from different times in the past. When extracting *S* from past climate reconstructions, it is necessary to *correct* *S* ^*p*^ for slow feedback processes. If this is carefully done [21], then the range of climate sensitivity values found from palaeoclimate studies of the last 65 million years (Myr) broadly confirms the range covered by mostly climate model and observation-derived values given by the IPCC [3]; at the 68 % probability level, the IPCC range is *S* = 0.4−1.2 K (W m ^−2^) ^−1^, while the PALAEOSENS approach has led to *S* = 0.6−1.3 K (W m ^−2^) ^−1^. On the one hand, this result is promising if we view the fact that independent estimates of climate sensitivity do, in principle, improve our confidence in the mean value of climate sensitivity. On the other hand, it is disappointing that the extreme values of the currently accepted range of possible climate sensitivity could not be more confidently rejected from inspection of the palaeoclimate records. This state of affairs has been explained by the uncertainty in palaeoclimate reconstructions (temperature changes, forcing, and feedback strengths), and the fact that *S* focuses only on global mean quantities, which are inherently difficult to determine from local proxy observations [11]. However, it may also be that the concept of climate sensitivity should be extended or generalised in order to better account for the spatial distribution of temperature and radiative forcings, and for the different factors that affect the definition of the climate sensitivity parameter given in Eq. 1 as has been already suggested by Skinner [26].

For example, the astronomical forcing does not fit the climate sensitivity definition (Eq. 1) because this forcing mainly influences climate through changes in the seasonal and latitudinal distributions of the insolation, with little direct effects on the global average [27]. Effects associated with the slow tectonic forcing and erosion may also alter both the mean state of climate and the climate sensitivity since feedbacks are generally expected to vary with the background climate [1, 15, 20, 23, 28–32]. More generally, the ratio (Eq. 1) between temperature and forcing may depend on the nature and spatial distribution of the forcing itself [26], as we clarify below. Finally, climate changes in the past as well as the present are a non-stationary response of the climate system to forcing and the question is, which, if any, of the responses can be considered to be in equilibrium. In past climate changes, a multitude of timescales (in forcing and response) play a role, and we cannot a priori assume a (constant) separation of fast and slow timescales. Even if there was timescale separation, non-linear processes in the climate system will introduce variability on any (new) timescale, which further complicates the analysis of the response to forcing in such a system. Estimating climate sensitivity from past climate changes (in models or observations) therefore requires a careful definition of the response timescale, the corresponding averaging procedure, and a careful analysis of the feedbacks involved. The presence of multiple scales in the response of the climate system to perturbations is apparent when one approaches the problem from the point of view of dynamical system theory and non-equilibrium statistical mechanics [33, 34].

The aim of this paper is to further clarify how the geological record can contribute to estimates of equilibrium climate sensitivity and its uncertainty. The focus is on the background state dependence of feedback processes and on the impact of tipping points on the climate system. Alternative concepts of climate sensitivity characterising the response to the present-day forcing in the complex climate system are also reviewed.

## Concepts

When determining *S*, it is mostly assumed that temperature differences are relatively small in comparison with the background temperature such that Δ*R* can be well approximated by

2 $$ {\Delta} R \simeq \left( \frac{\partial R}{\partial T}+ \frac{\partial R}{\partial \alpha}\frac{\partial \alpha}{\partial T}\right){\Delta} T.  $$

Here, the terms in brackets represent the Planck response ($\frac {\partial R}{\partial T}$) and the combined effect of all (net positive) feedback processes *α*(*T*) ($\frac {\partial R}{\partial \alpha }\frac {\partial \alpha }{\partial T}$) [35]. While the higher order terms in Δ*T* are usually small, they can become important and may lead to runaway climates, i.e. rapid climate change when the system crosses a tipping point, reinforced by positive feedbacks until a new steady state is reached [36, 37].

In [21], feedback processes *α* were divided into two categories: fast process *α* ^*f*^ with timescales smaller than *τ* and the slow process *α* ^*s*^ with timescales larger than *τ*. The radiative heat flux changes due to the slow processes *α* ^*s*^ have no effect on Δ*T* on the timescale *τ*, and hence, only the fast processes contribute to the radiative heat flux changes responsible for Δ*T*. In view of the present-day climate change, we select *τ* = 100 years (as suggested in [21]) and define

3 $$ S = \frac{\Delta T}{\Delta R_{[CO_{2}]}},  $$

where the total response Δ*T* (due to all fast processes with respect to the timescale *τ*) is measured with respect to the radiative heat flux change due to the change in atmospheric CO_2_. By including an arbitrary number *N* of fast processes in addition to the Planck response, *S* is given by

4 $$ S = \frac{-1}{\lambda_{P} + {\sum}^{N}_{i=1} {\lambda^{f}_{i}}}, $$

with the Planck response parameter *λ*_*P*_ < 0 and ${\lambda ^{f}_{i}} = {\Delta } R_{[{\alpha ^{f}_{i}}]}/{\Delta } T$ the feedback parameters of the fast process ${\alpha ^{f}_{i}}$. Using the surface energy balance, it was shown that *S* can be obtained from *S* ^*p*^, according to

5 $$ S = S^{p} (1 + \frac{{\sum}^{M}_{j=1} {\lambda^{s}_{j}}}{\lambda_{P} + {\sum}^{N}_{i=1} {\lambda^{f}_{i}}}),  $$

where each ${\lambda ^{s}_{j}} = {\Delta } R_{[{\alpha ^{s}_{j}}]}/{\Delta } T$ represents the feedback parameter of the slow process ${\alpha ^{s}_{j}}$ [21].

For practical estimates of *S*, all slow processes (with respect to the 100-year timescale) are considered forcings. Next, the specific climate sensitivity parameter

6 $$ S_{[CO_{2}, {\alpha^{s}_{1}}, \cdots, {\alpha^{s}_{m}}]} = \frac{\Delta T}{ {\Delta} R_{[CO_{2}]} + {\sum}^{m}_{j=1} {\Delta} R_{[{\alpha^{s}_{j}}]}},  $$

is computed leading to (if in reality there are *M* slow processes)

7 $$ S = \lim_{m \rightarrow M} S_{[CO_{2}, {\alpha^{s}_{1}}, \cdots, {\alpha^{s}_{m}}]}. $$

This approach is fully compatible with linear response theory [38, 39] where a linear relation is obtained between forcing and response although the equilibrium states are fully determined by non-linear processes. However, by linearly regressing the decay of the radiative imbalance on Δ*T* [23], feedback processes are assumed to stay constant, while deviations from the linear relationship have been suggested more recently on century timescales [40, 41], implying that the non-linear version of the response theory should be taken into account [34]. Moreover, interannual to decadal climate variability is generally averaged out by determining *S* intrinsically assuming that the processes generating the variability do not significantly interact with the background climate [33]. The definition of *S* in Eq. 3 allows for a consideration of the climate response (on the 100-year time scale) in any climate state of the Earth’s history including the present climate. Because the feedback processes need not be equally strong during all times, we specify a point in time to *S*, i.e. *S*(*t*), where *S*(*t* = 0) represents the present equilibrium climate sensitivity parametre (termed *S* ^*a*^ or ‘actuo’ climate sensitivity in [21]). The variation of *S*(*t*), *S*_[*X*]_(*t*), and *S* ^*p*^(*t*) over time is directly associated with the background state (or temperature) dependence of the feedback parametres *λ*_*P*_, *λ* ^*f*^, and *λ* ^*s*^, which we discuss in the next section.

## State Dependence of Climate Sensitivity

In this section, we review progress in understanding the state dependence of the climate sensitivity parameter *S*(*t*) from both palaeo data and modelling studies.

Using palaeo data to reconstruct climate sensitivity has the advantage of portraying real-world responses that integrate all (known and unknown) processes. A key drawback, however, is that the reconstructions rely on indirect (proxy) measurements of the relevant quantities. Many proxies rely on calibrations in the modern environment. Hence, their use into deep geological time may be complicated by subtle changes through time in overall ocean chemistry, uncertain extrapolation into environmental conditions outside the calibration window (non-analogue conditions), and chronological uncertainties between the various records that are being compared. Modelling studies can help tackle some of such potential complications, but in turn may be compromised by explicit and implicit assumptions, and by a potential lack of (or incomplete representation) of processes, whether known or unknown. Palaeoclimate sensitivity studies need to account for both observations and modelling results, including transparent consideration of uncertainties wherever possible. This is particularly important when addressing the subtle problem of potential state dependence, which we focus on here.

### State Dependence From Palaeo Data

**Fig. 1:**
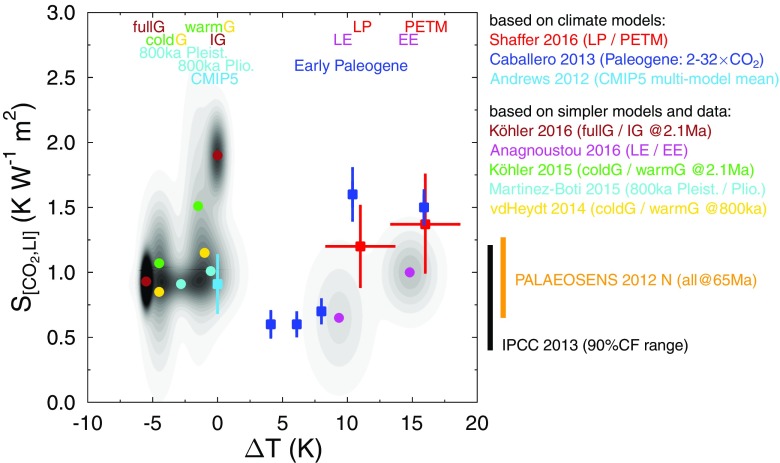
Published paleo-based values of $S_{\mathrm [CO_{2},LI]}$ (specific equilibrium climate sensitivity parameter caused by CO_2_ radiative forcing and corrected by variations in land-ice (LI) feedbacks) indicating its state dependence. Only studies published after the PALAEOSENS review paper [21] are considered. For comparison, the state-independent values from PALAEOSENS, and from the IPCC report [3], and the CMIP5 multi-model mean for present day [41] are also shown. All values of $S_{\mathrm {[CO_{2},LI]}}$ were given as mean (*or most likely*) ±1*σ*, apart from IPCC, which is the 90 % confidence (CF) range. Climate background states are given by Δ*T* from pre-industrial and are marked as estimated ranges (or ±2*σ*). In [42], further corrections for other slow feedbacks have been calculated, which has been ignored here, leading to different values of Δ*T* than published. To increase the clarity of the figure, the data-based results are visualised by *colour-coded circles* (mean values), while their uncertainties are combined in a cumulative probability density distribution (*grey shading*) assuming normal distributed values. Results based on climate models are shown by *colour-coded squares* (mean) including their uncertainties (*bars*). *G* glacial, *IG* interglacial, *LE* late Eocene, *EE* early Eocene, *LP* pre-PETM/late Paleocene, *PETM* Paleocene-Eocene thermal maximum. Reference numbers of the given citations: IPCC 2013 [3], PALAEOSENS 2012 [21], Andrews 2012 [41], Caballero 2013 [43] vdHeydt 2014 [20], Martinez-Boti 2015 [44] Köhler 2015 [32], Anagnoustou 2016 [42], Köhler 2016 [45], and Shaffer 2016 [46]

Some new (re-)interpretation of existing palaeo data has emerged since the review of the PALAEOSENS project in 2012. Previous observation-based studies [21, 47] had indicated that climate sensitivity might be state dependent, but we’re not yet able to quantify this dependence. Progress was made when it was inferred from data through the entire interval covered by ice cores (last 800 kyr) that *S* may have been 30–40 % lower during full glacial conditions than during intermediate glaciations [20] (see yellow dots in Fig. 1). This was found to apply to both the simplest version of the specific climate sensitivity parameter, *S*_[*CO*2,*LI*]_, in which CO_2_ radiative forcing was corrected only for the land-ice (LI) albedo feedback, and for the most complex version that also accounted for radiative forcing by all main greenhouse gases (GHGs), including CH_4_ and N_2_O, and including corrections for the slow feedbacks of albedo variation caused by vegetation (VE) and aerosols (AEs) (i.e. *S*_[*GHG*,*LI*,*VE*,*AE*]_). The general shape of this dependence was recently supported by a combined model-data approach [48]. Further support for a lower *S*_[*CO*2,*LI*]_ during colder periods was found [32, 45] (green/brown dots in Fig. 1), showing that an extension of this state-dependence analysis is possible over the last 2.1 Myr based on existing boron-based CO_2_ proxy data. Furthermore, ice sheet volume and area are explicitly calculated using an ice sheet model implying a non-linear relation between volume and extent essentially caused by the rheological characteristics of ice. These results suggest that the latitudinal variation and strength of the land-ice albedo feedback is important for detecting state dependence in *S*; hence, other approaches did not detect the state dependence, given that they approximated land-ice albedo feedback by more simplified means (e.g. based on sea-level changes [49]). In particular, Rohling et al. [27] estimated zonally averaged changes in the land-ice albedo feedback from a combination of sea-level change, annual mean incoming solar radiation, and area-weighted scaling factors accounting for the latitudinal distribution of ice sheets but nevertheless detected no significant state dependence.

A new dataset of proxy-based CO_2_ from the Pliocene [44] has been used to state that *S*_[*CO*2,*LI*]_ was similar in the late Pleistocene and the Pliocene implying no state dependence for *S* within the uncertainties (cyan points in Fig. 1). However, this study did not search for the state dependence of *S* within the Pleistocene itself and also used a sea level-based land-ice albedo feedback calculation, which would limit the potential to detect state dependence. The polar amplification factor is another uncertain parameter in these studies that could mask state dependence; climate models suggest a non-constant polar amplification and potentially less strong in the Pliocene than in the Pliocene [24, 32, 50, 51].

### State Dependence on Climate Models

There is a growing modelling literature on how climate sensitivity may change with the climate state. Early work with a simplified coupled climate model [52] indicated higher sensitivity in climates colder than modern, which they argued was due to an increased role for cryospheric surface albedo feedback. The same conclusion was drawn from a range of modern climate models [53]. A study using the GISS Model E confirmed higher sensitivity in colder climates but also found higher sensitivity at high forcing, with a minimum in sensitivity near the modern climate [54]. Recent work using a modified version of the same model supported this non-monotonic U-shaped behaviour of climate sensitivity [55]. None of these studies quantitatively analysed the underlying feedback mechanisms driving the changes in sensitivity. The first study to do so across a broad range of climates was using the Australian Bureau of Meteorology model spanning a range of 1/16 to 32 times modern CO_2_ [56]; they found monotonically decreasing sensitivity as CO_2_ and temperature increased via a combination of decreasing surface albedo and cloud short-wave (SW) feedback strengths. On the other hand, Caballero and Huber [43] conducted Eocene simulations using NCAR CCSM3 spanning 1 to 32 times CO_2_ and found a transition to much higher sensitivity when global mean temperature increased above 23 ^∘^C, driven by a sharp increase in positive SW cloud feedback and a more modest increase in water vapour feedback (see blue squares in Fig. 1). Within the same model [57] and the MPI-ECHAM6 over a smaller range of forcing [58], increasing sensitivity at higher CO_2_ was found due to mostly increased water-vapour feedback. As commonly found in model intercomparisons, the intermodel differences in sensitivity are driven mostly by cloud feedbacks. However, enhanced sensitivity in cold climates due to stronger surface albedo feedback and in warm climates due to stronger water vapour feedback emerge as robust features across models, suggesting that the non-monotonic U-shaped structure of climate sensitivity may have some qualitative validity (see Fig. 1).

Another line of work has focused on comparing the sensitivity to negative radiative forcing under last glacial maximum (LGM) conditions (due to combined decrease in CO_2_ and increased surface albedo) to positive forcing under doubled CO_2_ [15, 31, 59, 60]. These studies generally find a more muted response to negative than to positive forcing. This implies a weaker sensitivity for climates on the cold side of modern than on the warm side as the observation-based studies also suggest (which in turn implies that if the overall non-monotonic structure discussed above is true, then the minimum sensitivity is attained in climates colder than the LGM). However, these conclusions rely on an implicit assumption that a unit of radiative forcing by ice-sheet albedo is interchangeable with a unit of radiative forcing by CO_2_ [61]. This caveat also applies to the observation-based studies discussed above, and it is a key issue that remains to be tested [32].

## Climate Sensitivity in the Presence of Tipping Points

It has been well-recognised that the concept of equilibrium climate sensitivity is quite limited when making adequate projections of global mean surface temperature for the end of this century [33]. The climate system has a strong internal variability on many timescales, is subject to a non-stationary forcing, and certainly is out of equilibrium with the changes in the radiative forcing up to the year 2100. Moreover, in the past, abrupt shifts have occurred, and also for the present climate, a number of potential tipping elements have been identified [66, 67].

**Fig. 2:**
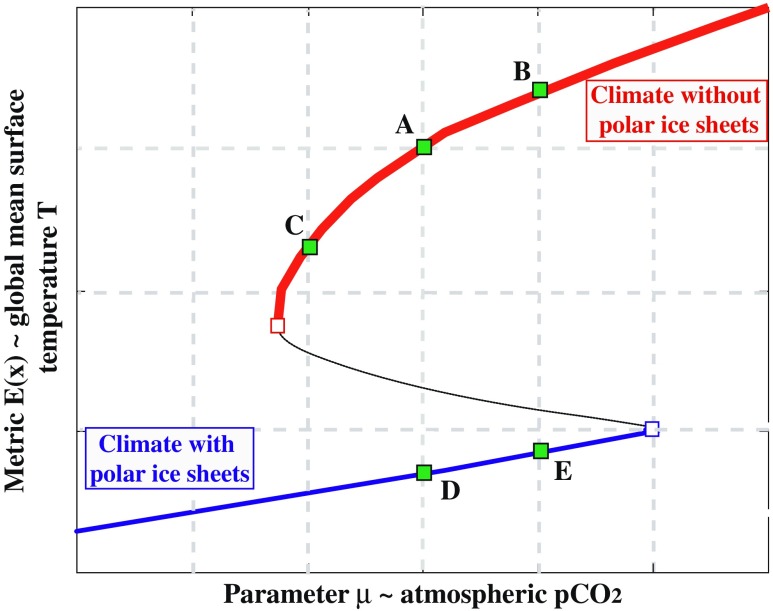
Schematic of the phase diagram of a climate model with two stable coexisting climate states. The shape of the S curve follows closely that discussed in [62–64]; see also [65]. The climate sensitivity parameter *S* is defined on each of the stable branches as the local slope of the global mean surface temperature *T* versus the (logarithm of) atmospheric pCO_2_ (cf. Eq. 8). Type I state dependence: When starting at *point A* (e.g. the pre-industrial climate), the temperature increase after a doubling of pCO_2_ (*point B*) is smaller than when starting from a colder climate (*point C*) on the same branch. Type II state dependence: When the initial pCO_2_ is the same as in *point A*, but the climate is initially on the cold branch (*point D*), a doubling of pCO_2_ results in a smaller temperature increase (*point E*) than if starting from *point A* and ending in *point B*. *S* becomes undefined at the transition points (*open squares*) between the two branches. The conditional climate sensitivity is equal to *S* for small perturbations (going from *points D* to *E*), but largely increases if the perturbation in CO_2_ is large enough to move the system from *point D* beyond the bifurcation point (*blue open square*) and jumps to the warm branch. Note that *S* is generally defined as a local gradient, while the 2xCO_2_ definition in the ECS may involve a perturbation too large for the linear assumption along the branch to be applicable

Consider, for example, a typical zero-dimensional energy balance model as shown in [68], which has for a range of atmospheric CO_2_ concentrations two stable climate states coexisting, an ice-free state and an ice-covered one (see Fig. 2). These two climate states are often interpreted as the snowball Earth and a much warmer greenhouse climate, which both may have existed in the far distant past [69, 70]. There can be, however, more branches in such a system [71], including partially glaciated climate states. The transition from the greenhouse climate during the early part of the Cenozoic to the climate states with major polar ice sheets (on one or both hemispheres) that existed since the Oligocene [72] can, therefore, conceptually be considered as a transition between different branches like those in Fig. 2.

The equilibrium climate sensitivity *S* is well defined along most of the two branches, but infinite at the bifurcation (tipping) points themselves. Moreover, the local slope along each branch depends on how feedback processes depend on the background temperature, i.e. it does not need to be constant. As long as there is a steady-state branch, the climate sensitivity parameter *S* (Eq. 1) is given by

8 $$ {S} = \frac{\partial E(\textbf{x}(\mu))}{\partial R_{[\mu]}},  $$

where *E*(**x**) is a metric for the state of **x** (i.e. the global mean temperature *T*) and the parameter *μ* indicates the (logarithm of the) CO_2_ concentration (see Fig. 2).

These considerations lead us to define two types of state dependence of climate sensitivity. Type I refers to the fact that the strength of fast feedbacks depends on the control parameter, so that *S* on one branch varies with *T*. To define the type II state dependence, let us first observe that at the (saddle node) bifurcation points (open squares in Fig. 2), the derivative becomes infinitely large, indicating a structural change in the model climate system. This structural change is reflected in the two branches of the system, which exhibit different behaviours as to climate sensitivity: at one particular pCO_2_ in the multiple equilibria range, two (different) values for *S* can be determined. If the slopes on the two branches are different, then the climate sensitivity depends which branch the system is on. In other words, the climate sensitivity depends on the active feedbacks determining the background climate. The type II state dependence then refers to the fact that the strength and number of active feedbacks may depend on the equilibrium point being visited, even if the control parametre is the same. As the saddle-node bifurcation is approached, non-linear effects may become increasingly important, such that the first-order Taylor expansion of the radiative forcing (see Eq. 2) may not be sufficient any more. The closer to the bifurcation point the background climate is, equilibrium non-linearity leads to run-away climate, or at least jumps to some other stable climate branches [37].

In [68], the concept of conditional climate sensitivity parametre *S*(*δ*,*t*_*e*_) of a background climate state (indicated by $\bar {T}$) was defined as

9 $$ S(\delta, t_{e}) = \frac{\Delta T(\delta, t_{e})}{\Delta R(\delta, t_{e})}  $$

where ${\Delta } T(\delta , t_{e}) = | T(t_{e}) - \bar {T} |$ is the maximum temperature difference that can occur under the constraint $| T(0) - \bar {T} | < \delta $ over a time *t*_*e*_, and Δ*R*(*δ*,*t*_*e*_) is the change in radiative forcing over the same time interval. Note that scalar norms are used here, but the model to determine it can be very high dimensional. In a climate system in which there is a single equilibrium for each value of pCO_2_, *S*(*δ*,*t*_*e*_) is independent of *δ* (i.e. there is no region of conditional stability) and will approach the equilibrium climate sensitivity *S* parameter in the limit *t*_*e*_ → *∞*. In this limit, Δ*T* will be precisely the difference between the temperature of the equilibrated states and Δ*R* the difference in radiative forcing between both states (Eq. 8).

The conditional climate sensitivity is also suited for situations in which bifurcations occur (and type II state dependence). For example, suppose we are on the lower branch (point D in Fig. 2), when increasing CO_2_ (or *μ*) up to point E, the equilibrium climate sensitivity is relatively small. Such values are found in [68], for *t*_*e*_=100 years when *δ* is relatively small. When the initial perturbations are large (i.e. increasing CO_2_ beyond the blue open square in Fig. 2), however, the system may jump to another state (upper branch in Fig. 2), increasing the sensitivity greatly.

In addition to steady-state dynamics, more complicated dynamics may occur on each of the branches such as periodic orbits. Self-sustained oscillations mimicking the late Pleistocene ice-age cycles have been found in models by [73–79], and also in a slightly more complex model by [80] as relaxation oscillations. The issues of internal climate variability on palaeoclimatic timescales are discussed in more detail in [77, 81, 82]. In these cases, while *S* still is defined by Eq. 8, Δ*T* may not be a smooth function any more, critically depending on the timescale of interest. For example, when considering the Pleistocene ice ages as (long-term) oscillations, the sensitivity derived from comparing glacial with interglacial periods will differ from the sensitivity to CO_2_ doubling in a climate model, because the two extremes of the oscillation cannot be reached within a century timescale [83]. Shorter timescale variability may be represented by stochastic noise in the forcing, which then requires adaptation of climate sensitivity in probabilistic terms [84].

## Challenges Ahead

One of the main insights about climate sensitivity that has been gained from palaeoclimate studies is its potential state dependence. Consequently, findings about the value of *S* from past climate states that are colder (most of the Pleistocene) or significantly warmer (e.g. Eocene) may not be directly applicable to projections of global warming in the present climate due to anthropogenic CO_2_ emissions. Nevertheless, for future climate projections, it is crucial to know whether there is state dependence or not and which processes are responsible. This is particularly important for improving the confidence in climate models used for future projections, if processes such as cloud feedbacks are involved that are not adequately captured in those models [85]. The challenge now is to further quantify state dependence from past climate changes and to understand the responsible processes from both models and data.

On the data side, adequate statistical methods should be used to detect whether scatter plots of temperature change over radiative forcing (Δ*T* over Δ*R*) follow (locally) linear or non-linear behaviour indicating constant or state-dependent characteristics of *S*_[*X*]_. More precise and higher resolved CO_2_ data (for the pre-ice core interval > 800 kyr) and more robust time series of global temperature change as well as (model-interpreted) land-ice and vegetation reconstructions will be necessary to quantify the state dependence of *S*.

The difficulty lies, however, not only in the uncertainties intrinsic to proxy reconstructions: even if we were able to reconstruct, say, past CO_2_ levels and ice sheet extents with great accuracy, there would remain considerable uncertainty in the radiative forcing actually supplied. Recent work [41] quantifying the forcing due to quadrupled CO_2_ across the CMIP5 model ensemble, for example, shows that the canonical value of 3.7 Wm ^−2^ for CO_2_ doubling is precise only to about 1 Wm ^−2^. Much of this uncertainty comes from the role of tropospheric adjustment—the direct tropospheric response to CO_2_ before surface temperature has changed—whose importance has only recently come to the fore [61]. The uncertainty in radiative forcing due to ice sheets is even less well known, having been quantified in only a handful of models (e.g. [60, 86, 87]). The treatment of CO_2_ as forcing in the climate sensitivity framework needs further clarification; in past climate changes, co-variation is a dynamic process [83, 88] while in models used for future projection the response to fixed CO_2_ is considered. Finally, potentially differing efficacies of forcings remain largely unexplored. Orbital forcing [89] is generally believed to play a key role in the waxing and waning of glacial cycles [90, 91], although the precise mechanisms remain debated [92–94]. Feedback processes in particular those related to snow, sea-, and and land-ice albedo [47, 95] are central to understanding ice age cycles. Most likely, the annual mean radiative imbalance caused by these feedback processes is much stronger than the initial orbital radiative forcing [27, 32, 49]. In climate sensitivity studies, orbital forcing has been largely ignored because the direct effect of forcing is predominantly seasonal (at least the precessional component), while the climate response is measured on century timescales focusing on the global annual mean value of the radiative forcing [21]. However, when determining climate sensitivity in periods with glacial cycles, differing orbital configurations need to be taken into account. For example, climate sensitivity measured from the Eemian period, where summer insolation was high in the Northern Hemisphere, is difficult to compare with the recent anthropogenic warming caused by enhanced levels of greenhouse gases. This difference is only partly reflected in the state dependence, as seen in the different values of *S* during glacial and interglacial periods [20, 32, 96]. Disentangling the contributions to *S* of orbital forcing and feedback state dependence requires running climate models under different orbital configurations, which at the same time effectively performs out-of-sample tests of the models [97]. Even outside the glacial-interglacial cycles, it is important to quantify how orbital variations affect local proxy data [98].

Another problem still lies in the quantification of *S* from time series of palaeo data. Recent work [45] suggests that the methods for calculating *S* can not simply be transferred from the state-independent to the state-dependent case, because the state dependence may appear as a non-smooth (not even locally) relationship between *S* and *T*. A further complication arises when tipping points have been crossed in the time series [20], where the classical definition of *S* breaks down. Therefore, robust methods to detect and interpret those tipping points in palaeo data need to be further developed [99–101], but also the recently suggested generalisations of *S* such as the conditional climate sensitivity [68] or the quantification of non-linear contributions in the Taylor expansion of the radiative balance [37] need to be further explored.

When it comes to alternative metrics for characterising the response of the climate system to changes in the forcing, clearly, more than one single climate sensitivity parametre is required. The difficulty of accounting for the effect of orbital forcing highlights two major shortcomings in the concept of climate sensitivity, namely the lack of both spatial and temporal structures in the climate response.

Not only the radiative forcing varies in latitude but also the temperature response may exhibit spatial structure, e.g. [27]. One important and still a large-scale quantity is the equator-to-pole temperature gradient, which is generally believed to be smaller in the ice-free and high greenhouse gas climates of the early Cenozoic with different feedbacks at work [102–105]. In colder climate states with ice, the temperature response is clearly latitude dependent as reflected in polar amplification factors between polar and global mean temperature change. Polar amplification varies over time, but how remains largely unknown [32, 59, 60, 106, 107]. Another form of spatial and temporal patterns in the response to forcing can appear in terms of the major modes of climate variability such as the El Ni˜no Southern Oscillation (ENSO) or the monsoons, which may change their pattern, amplitude, or frequency through (non-linear) interactions with the background climate [108–111].

An extension of the concept of *S* as given by Eq. 8 to the non-stationary nature of the climate response could be to also consider the change in higher order moments (e.g. variance, skewness) and the long tails of distributions of temperature instead of only using the (temporal) mean as metric *E*(**x**). The information contained in all moments together can be captured in one single metric (Wasserstein distance) as suggested recently in a non-autonomous stochastic dynamical system approach to climate sensitivity [33, 112]. Response theory provides an additional way for incorporating rather general measures of climate sensitivity; it allows prediction of both globally averaged quantities and spatial patterns of change [39]. In all of these approaches, the challenge is to keep concepts simple enough to be applied to palaeo data without oversimplification.

In conclusion, while a major challenge remains to improve accuracy in past climate reconstructions, one of the important issues we can learn from past climates about climate sensitivity is to better understand its potential state dependence in order to eventually reduce the uncertainty in ECS. At the same time, from a theoretical point of view, it is necessary to further develop and apply alternative concepts for quantifying the response to forcing in the highly complex climate system as have been outlined in this review, with special emphasis to those, which can also (practically) be determined from the geological record.
